# Passive immunoprophylaxis and therapy with humanized monoclonal antibody specific for influenza A H5 hemagglutinin in mice

**DOI:** 10.1186/1465-9921-7-126

**Published:** 2006-10-14

**Authors:** Brendon J Hanson, Adrianus CM Boon, Angeline PC Lim, Ashley Webb, Eng Eong Ooi, Richard J Webby

**Affiliations:** 1 Defence Medical and Environmental Research Institute, DSO National Laboratories, 27 Medical Dr., Singapore 117510; 2 Department of Infectious Diseases, St Jude Children's Research Hospital, 332 N Lauderdale, Memphis, TN38103, USA

## Abstract

**Background:**

Highly pathogenic avian H5N1 influenza virus is a major public health concern. Given the lack of effective vaccine and recent evidence of antiviral drug resistance in some isolates, alternative strategies for containment of a possible future pandemic are needed. Humanized monoclonal antibodies (mAbs) that neutralize H5N1 virus could be used as prophylaxis and treatment to aid in the containment of such a pandemic.

**Methods:**

Neutralizing mAbs against H5 hemagglutinin were humanized and introduced into C57BL/6 mice (1, 5, or 10 mg/kg bodyweight) one day prior to-, one day post- and three days post-lethal challenge with H5N1 A/Vietnam/1203/04 virus. Efficacy was determined by observation of weight loss as well as survival.

**Results:**

Two mAbs neutralizing for antigenically variant H5N1 viruses, A/Vietnam/1203/04 and A/Hong Kong/213/03 were identified and humanized without loss of specificity. Both antibodies exhibited prophylactic efficacy in mice, however, VN04-2-huG1 performed better requiring only 1 mg/kg bodyweight for complete protection. When used to treat infection VN04-2-huG1 was also completely protective, even when introduced three days post infection, although higher dose of antibody was required.

**Conclusion:**

Prophylaxis and treatment using neutralizing humanized mAbs is efficacious against lethal challenge with A/Vietnam/1203/04, providing proof of principle for the use of passive antibody therapy as a containment option in the event of pandemic influenza.

## Background

With the initial outbreak of pneumonia caused by highly pathogenic H5N1 influenza A virus in Hong Kong in 1997 resulting in the death of 6 of the 18 infected individuals [1,2], the potential for emergence of a pandemic influenza was recognized [3]. Mass slaughtering of poultry appeared to halt the outbreak. However, H5N1 viruses of various genotypes have spread across Southeast Asia and have continued to cause disease outbreaks in poultry and aquatic birds. Toward the end of 2003, a single genotype (the "Z-genotype") became dominant and was responsible for outbreaks in Indonesia, Thailand and Vietnam throughout 2004 [4], causing death in ~50% of the confirmed cases.

Since this time, the H5N1 influenza epidemic of the Asian bird population has continued. In addition, infection of migratory birds has resulted in increased global spread of the virus with reports of H5N1 influenza causing mortality in poultry and aquatic birds throughout Asia, Europe and Africa. From the beginning, the ability of H5N1 viruses to cross the species barrier was evident not only from the cases of human infection, but also from infection and mortality in domestic cats, captive tigers and leopards [5,6]. Taken together, the increased incidence of human infection, coupled with evidence of expanding host range and widespread distribution of H5N1 viruses has heightened concern that acquisition of the properties necessary, through mutation or genetic reassortment, for human-to-human transmission is only a matter of time. Should this occur, humans would have virtually no immunity to such novel viruses which may result in a human influenza pandemic of potentially catastrophic proportions [7].

Currently, control of influenza relies on two options, vaccination or antiviral drug treatment with vaccination being the preferred option. However, the high virulence of H5N1 influenza has inhibited the development of vaccines using traditional approaches [8]. Other approaches for vaccine production, such as reverse genetics, DNA vaccination and the use of recombinant hemagglutinin [8-10], have been met with varying degrees of success but a human vaccine ready for commercial production is still not available. Therefore, should a pandemic arise due to H5N1, the lack of an effective vaccine means containment would rely solely on the effectiveness of antiviral drugs and physical measures to inhibit viral spread such as social distancing.

For treatment of influenza two classes of antiviral drugs are presently licensed: M2 ion channel inhibitors (amantadine and rimantadine) and neuraminidase inhibitors (NAIs; oseltamivir and zanamivir). The presence of H5N1 viruses resistant to the M2 inhibitors [4,11], means their use cannot be relied upon. The neuraminidase inhibitors are currently viewed as the best choice for prophylaxis against- and clinical management of- disease due to H5N1 virus. Its efficacy in both uses is still unclear due to a lack of human data. Recent studies in mice have highlighted prophylactic efficacy against infection by H5N1 virus isolated from Vietnam [12]. However, reports have also appeared detailing the development of oseltamivir resistance during treatment of H5N1 infected patients and isolation of drug resistant H5N1 virus in the same region [13,14]. While the current data does not deter stockpiling of oseltamivir for pandemic response [15], it does suggest that alternative strategies for prophylaxis or treatment are warranted.

Passive immunization may provide an alternative strategy for both prophylaxis and treatment against pandemic influenza. For a number of viral diseases, such as rabies, hepatitis and respiratory syncytial virus (RSV), administration of antibodies derived from hyper-immune sera of human or animal origin have been used effectively as either prophylaxis or treatment in high risk individuals where vaccination was not possible [16]. Indeed previous studies have shown the effectiveness of neutralizing antibodies specific for hemagglutinin as both a treatment for established influenza A infection and prophylaxis against influenza virus challenge in a mouse model [17-19]. Recently, equine hyperimmune globulin F(ab')2 has been shown to effectively treat influenza A H5N1 virus infection in a mouse model [20], however use of animal derived antibodies can result in severe anaphylactoid side effects [21] and the induction of human anti-species specific antibody responses which limits the efficacy of the antibodies with repeated use. We report here, the protective efficacy of neutralizing humanized monoclonal antibodies specific for the hemagglutinin of a Z-genotype influenza A H5N1 virus in mice when used as prophylaxis before-, and treatment following-lethal challenge with fully pathogenic H5N1 virus.

## Methods

### MAbs to Hemagglutinin of A/Vietnam/1203/04 (H5N1)

MAbs to the HA of A/Vietnam/1203/04 and A/Hong Kong/213/03 were prepared in mice immunized with attenuated versions of the respective H5N1 virus generate by reverse genetics using a modification of the method described by Kohler and Milstein [22,23].

### Construction of human IgG1 constant region expression vector

Design of the expression vector for human IgG1 was based on that described by Jostock et al, 2004 [24]. Briefly, a synthetic construct containing the recognition sites for *ApaL1, Pst1, Asc1, Nco1, Mfe1, Xho1 *and *Xba1 *with a synthetic secretion leader sequence between *Nco1 *and *Mfe1 *was used to replace the multiple cloning site, myc-tag and ER retention signal of pCMV/myc/ER (Invitrogen). The internal ribosome entry site (IRES) of encephalomyocarditis virus was inserted between *Asc1 *and *Nco1 *following amplification from pIRES (Clontech) to introduce the relevant sites. For insertion of the human antibody constant regions, cDNA clones (I.M.A.G.E. Consortium cDNA clones [25]) encoding the Kappa light chain (Clone ID 6279986) and the IgG1 heavy chain (Clone ID 6281248) were amplified to allow insertion of the constant regions between *Pst1 *and *Asc1*; and *Xho1 *and *Xba1*, respectively. Recognition sites within the antibody constant regions affecting cloning were removed by site-directed mutagenesis.

### Cloning of chimeric IgG1 expression vectors

To isolate the cDNA encoding the variable regions of the monoclonal antibodies, mRNA was prepared from hybridoma cells and used in first strand cDNA synthesis with random hexa-nucleotides. The total cDNA was then used as template in reactions to amplify both the variable heavy and light chain using the primers and protocols of the mouse scFv recombinant antibody phage system (Amersham Biosciences) with the resulting products cloned into pCR-Script (Stratagene) for sequencing. Variable region specific primers were used to amplify both the heavy and light chain variable regions with addition of recognition sites to allow cloning between the *Mfe1 *and *Xho1*; and *ApaL1 *and *Pst1 *sites of the human IgG1 constant region expression vector, respectively. Cloning according to this protocol produces constructs from which expression gives rise to chimeric antibodies containing the mouse variable and human constant regions.

### Transient expression of chimeric antibodies and purification

Chimeric antibodies were expressed using the FreeStyle™ 293 expression system (Invitrogen) to obtain antibodies produced in a defined, serum-free medium. Constructs encoding chimeric IgG1 were transfected into 293-F cells by use of 293 fectin (Invitrogen). Supernatants were collected 120 h after transfection and proteins purified using protein A sepharose beads (Amersham). Purity of IgG was confirmed using SDS-PAGE analyses. ELISA using HRP labeled anti-mouse IgG (Sigma) and anti-human IgG (Accurate Chemical & Scientific Corporation) was used to highlight the introduction of human IgG constant regions

### Virus neutralization and HI tests

Virus neutralization tests were performed in Madin Darby canine kidney (MDCK) cells and hemagglutinin inhibition (HI) assays were performed with 0.5% chicken red blood cells as previously described [22]. In each HI assay four hemagglutinin units (HAUs) of virus were used and 100 50% tissue culture infective doses (TCID_50_) were used in each of the virus neutralization tests.

### Epitope mapping

Mapping of the epitope recognized by VN04-2 and VN04-3 was performed using HI assay data as an indication of antibody recognition, where high titer indicated strong binding and low titers indicates negligible or non existent binding. HI assay data from a recent paper that examined the antigenic properties of multiple H5N1 sublineages, using a number of mAbs against various H5N1 isolates including VN04-2 (referred to as 15A3 in reference [26]), was also included. Alignment of the H5 amino acid sequences was and scattered mutations in positions not common to all of the HI assay negative isolates were not included in the examination.

### Protection of mice with chimeric mAbs

All mouse studies were conducted under applicable laws and guidelines of and after approval from the St. Jude Children's Research Hospital Animal Care and Use Committee. Female 6–8 weeks old C57BL/6 mice (Jackson Laboratories) were housed 5 per cage in ABSL3+ containment. Food and water were provided *ad libitum*. Mice (5 per group) received the indicated amount of antibody eg 1, 5 and 10 mg/kg of bodyweight in approximately 300 μL of sterile phosphate-buffered saline (PBS) by intraperitoneal (IP) injection. The control group received 300 μL of PBS by IP. For lethal virus challenge, mice were inoculated intranasally with 10 MLD_50 _(50% mouse lethal dose) in 30 μL of PBS of a fully virulent genetic clone of A/Vietnam/1203/04 virus derived by reverse genetics. This virus is highly pathogenic in mice without prior adaptation and symptoms preceding death are weight loss >30%, general inactivity and the development of hind leg paralysis. For the prophylaxis study, mice received the antibodies at the indicated doses 24 hours prior to lethal virus challenge. To determine therapeutic potential, mice were given a lethal virus dose, followed by the indicated amounts of antibody either one or three days post challenge. Morbidity and mortality were monitored for 21 days and the mice were weighed on days 4, 7, 10, 13 and 15 following virus challenge.

## Results

### Characterization of H5 neutralizing mAbs

Monoclonal antibodies against H5N1 viruses of the Z genotype which had shown high titers in HI tests against their respective immunogens were tested for their virus neutralizing capabilities. H5N1 viruses isolated from human cases throughout late 2003 and 2004 were known to differ in the antigenic loop located above the receptor binding site, with a potential glycosylation site in the latter [22]. Antibodies binding to this antigenic loop are neutralizing due to steric hindrance of the interaction between the receptor binding site of HA and its receptor located on the cell surface [27], glycosylation of this loop may inhibit binding of the antibody destroying it virus neutralizing properties. Therefore virus neutralization was performed with A/Hong Kong/213/03 in addition to A/Vietnam/1203/04 to allow identification of neutralizing mAbs that were not dependant on this region for activity. As highlighted in table 1 both VN04-2 and VN04-3 exhibited similar virus neutralization titers with both of the H5N1 isolates, while VN04-6 and HK03-3 did not. Therefore VN04-2 and VN04-3 were selected for humanization and efficacy studies in a mouse model.

**Table 1:** Virus-neutralization titers of mAbs against HA of H5N1

	mAb to H5N1 HA			
Virus	VN04-2	VN04-3	VN04-6	HK03-3

A/Vietnam/1203/04	512	>512	>512	256
A/HK/213/03	512	>512	12	>512

Virus neutralization assays were performed in MDCK cells. Titers are the reciprocal lowest dilutions of mAbs that completely inhibited 100 TCID_50 _of virus.

### Humanization of H5N1 neutralizing mAbs

**Figure 1:**
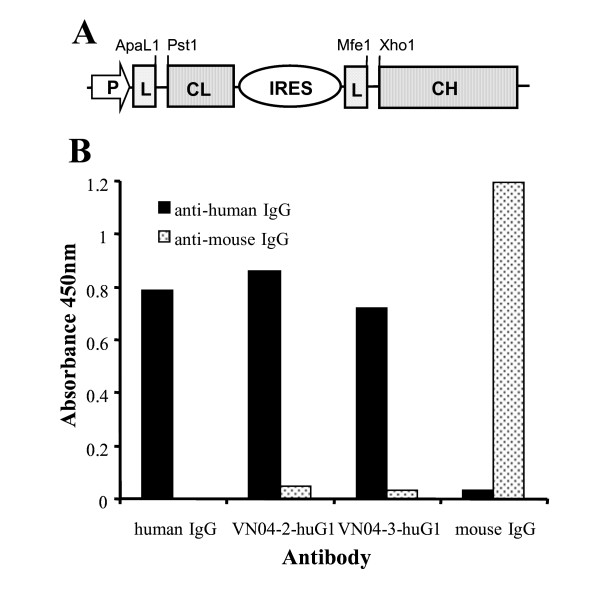
**Humanization of mouse mAbs VN04-2 and VN04-3**. *A*, Diagrammatic representation of the expression vector used to create chimeric antibodies; CL and CH refer to the constant regions of the human Kappa light and human IgG1, respectively; L refers to the leader sequence. *B*, ELISA to show presence of human constant regions, antibodies bound to immunosorbent plates were detected using secondary antibodies specific for human IgG and mouse IgG. Following addition of TMB substrate absorbance was measured at 450 nm.

To humanize the mAbs, we constructed chimeric antibodies where the coding regions of the mouse antibody variable domains were fused to the coding region of the constant domains of the human kappa light chain and IgG1 heavy chain using the construct described in figure 1A. Both chimeric antibodies (VN04-2-huG1 and VN04-3-huG1) were purified and their purity was confirmed by SDS-PAGE analysis, where resolution characteristics of the antibodies were observed and the preparation determined to be essentially free of contaminants (data not shown). Positive ELISA only in the presence of antibodies specific for human IgG confirmed the humanization of the mouse mAbs (figure 1B).

In order to ensure that humanization of the mAbs did not destroy specificity; we examined the performance of the humanized version of the antibodies in HI assays (table 2). For both VN04-2-huG1 and VN04-3-huG1, the titers for the assays against A/Vietnam/1203/04 and A/Hong Kong/213/03 indicated that the specificity was retained.

**Table 2:** HI assay testing of humanized mAbs against HA of H5N1

	mAb to A/Vietnam/1203/04 HA			
Virus	VN04-2	VN04-2-huG1	VN04-3	VN04-3-huG1

A/Vietnam/1203/04	6400	400	3200	800
A/HK/213/03	6400	3200	6400	3200

HI assays were performed in microtiter plates with 0.5% chicken RBC. Titers are the reciprocal lowest dilutions of antibodies that inhibited hemagglutination caused by 4 HAU of virus

### Prophylactic efficacy of VN04-2- and VN04-3 huG1 against A/Vietnam/1203/04 virus in vivo

**Figure 2:**
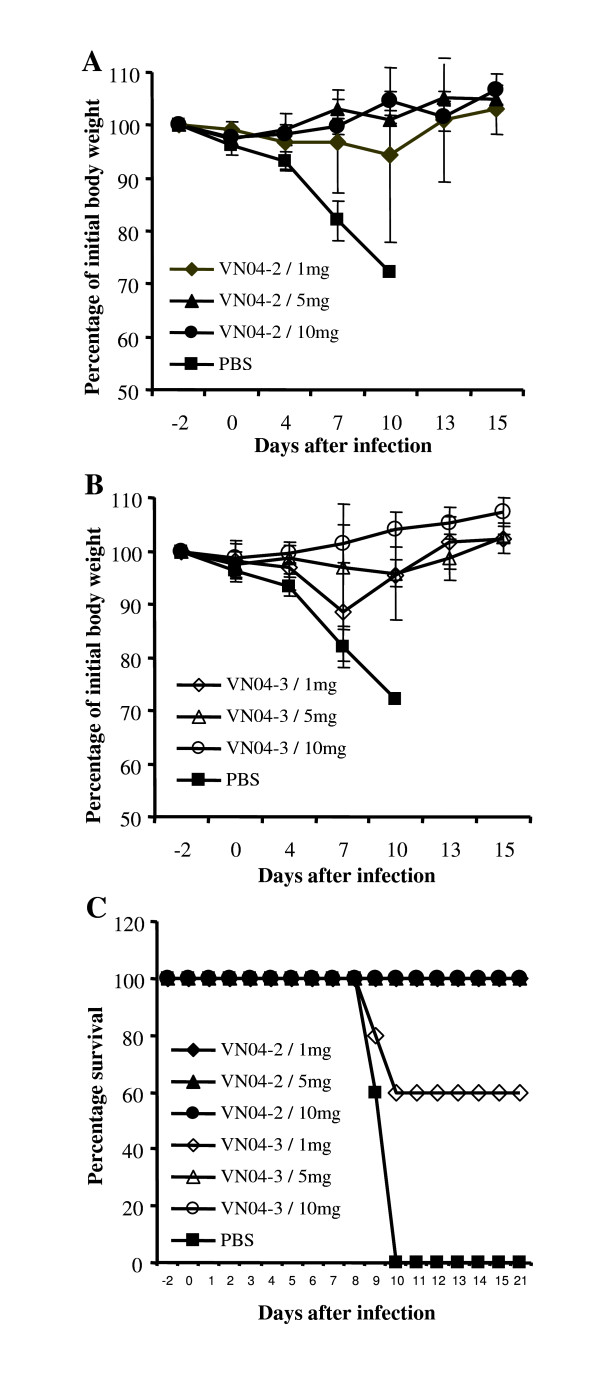
**Prophylactic efficacy of VN04-2- and VN04-3-huG1 in mice**. Mice were challenged with a lethal dose (10 MLD_50_) of fully virulent A/Vietnam/1203/04 24 h after the introduction of 1, 5, or 10 mg/kg bodyweight of antibody. The percentage of initial body weight after challenge is indicated for VN04-2-huG1 (*A*) and VN04-3-huG1 (*B*) periodically over 15 days. Each data point represents the average of 5 mice. Survival of challenged mice was observed for 21 days after challenge and indicates the level of protection from mortality (*C*).

To evaluate prophylactic efficacy of the chimeric antibodies, VN04-2-huG1 and VN04-3-huG1 were introduced into mice at the indicated doses twenty four hours prior to lethal virus challenge. Mice receiving low doses of VN04-2-huG1 antibody (1 mg/kg bodyweight) demonstrated few clinical disease signs including weight loss and death after virus challenge. Only one mouse lost more then 10% of its original bodyweight with full recovery by day 15 (figure 2A). Increased amounts of this antibody (5 or 10 mg/kg bodyweight) completely protected mice from disease upon challenge (figure 2A and 2C). Prophylactic efficacy was also observed for VN04-3-huG1, although not at the extent of VN04-2-huG1, as three mice receiving 1 mg/kg bodyweight showed significant weight loss of more then 10% (figure 2B), two of which were found dead by day 10 after virus challenge (figure 2C). Treatment with 5 mg/kg bodyweight of VN04-3-huG1 exhibited similar efficacy as with 1 mg/kg bodyweight of VN04-2-huG1. Finally, 10 mg/kg bodyweight of antibody VN04-3-huG1 completely protected mice from any clinical signs including death after challenge with H5N1 virus.

### Therapeutic efficacy of VN04-2-huG1 against A/Vietnam/1203/04 virus in vivo

**Figure 3:**
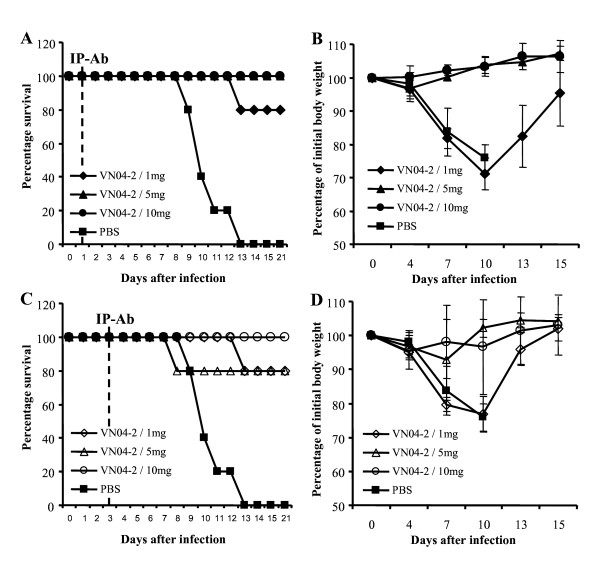
**Therapeutic efficacy of VN04-2-huG1 in mice**. Mice were inoculated with a lethal dose (10 MLD_50_) of A/Vietnam/1203/04 virus 24 h, followed by the introduction of 1, 5, or 10 mg/kg bodyweight of VN04-2-huG1 antibody one (*A and B*) and three (*C and D*) days post infection. The percentage of initial body weight was monitored periodically over 15 days (*B and D*) and each data point represents the average of 5 mice. Survival of mice was observed for 21 days following infection and indicates the level of protection from mortality (*A and C*).

Since VN04-2-huG1 showed greater prophylactic efficacy than VN04-3-huG1, therapeutic efficacy was determined for this antibody alone. The indicated dosages of antibody were introduced one and three days post lethal virus infection (figure 3). When the antibodies were given one day after infection (figure 3A and 3B), 1 mg/kg bodyweight of VN04-2-huG1 showed 80% protection, the remaining mice did show significant signs of disease but recovered by day 15. The higher doses of antibody (5 or 10 mg/kg bodyweight) completely protected the mice and showed little sign of disease. When antibodies were introduced three days after infection (figure 3C and 3D), 10 mg/kg bodyweight of VN04-2-huG1 was required to confer complete protection, with lower doses (1 and 5 mg/kg bodyweight) showing 80% protection. The lower dosages of antibody also showed increased signs of disease; however all of the mice that did not succumb to infection recovered the initial weight loss by day 15.

### Epitope mapping

To determine the epitope on the H5 hemagglutinin protein recognized by VN04-2 and VN04-3, we examined the amino acid differences between HAs of H5N1 viruses, using HI assay results as a measure of antibody binding (table 3). The majority of mutations occurred within the 140s and 150s antigenic loops identified from studies of H3N2 which are positioned direct below and directly above the receptor binding site, respectively [27]. Two positions were identified which differed in all of the HI assay negative isolates, amino acid 94 and amino acid 140 within the 140s loop. However the amino acid at position 94 faces the inside of the HA trimer and is unlikely to affect antibody binding. Therefore the epitope recognized by VN04-2 is most likely within or in close proximity to 140s antigenic loop, with residue 140 showing significant contribution to the specificity as even viruses with mutation only in this residue of the 140 loop were not inhibited by the antibody in HI assays. Other residues of the 140 loop may also be important for antibody binding as mutation at residues 138 and 141, as observed in A/Mdk/Jiang Xi/1653/05, also lowered the inhibitory effect of VN04-2 but not to the extent as did mutation of residue 140.

**Table 3:** Epitope mapping of VN04-2

Virus	HI Titer	Amino acid position in HA1							
		
				140s Loop			150s Loop		
		
		94	124	138	140	141	154	155	156
A/Vietnam/1203/04	+++	D	S	Q	**K**	S	N	S	T
A/Hong Kong/213/03	+++	.	.	.	**.**	.	.	N	A
A/Dk/Hong Kong/2986.1/00*	+++	.	N	.	**.**	.	.	.	A
A/Mdk/Jiang Xi/1653/05*	++	.	N	L	**.**	P	.	.	A
A/Mdk/Jiang Xi/2136/05*	-	N	D	.	**R**	.	.	N	A
A/Dk/Vietnam/568/05*	-	N	D	.	**N**	.	.	D	A
A/Qing Hai/05*	-	N	D	.	**R**	.	.	N	A

Viruses where HI assay titer data was taken from Chen et al, 2006 [26] are highlighted by asterix. Residues matching that in A/Vietnam/1203/04 are represented by full stop. Residue numbering refers to the position in A/Vietnam/1203/04.

## Discussion

In this study, we have reported the protective potential of two mAb specific for hemagglutinin of a Z-genotype H5N1 virus A/Vietnam/1203/04. Previous studies examining the antigenic sites of hemagglutinin of influenza A H3N2 have identified two protruding loops, residues 140–146 (140s loop) and 155–164 (150s loop), located near the receptor-binding site that are antibody-binding sites for potent neutralizing antibodies against this virus [28]. These loops are prone to antigenic drift and mutation introducing a potential glycosylation into this region inhibits antibody binding [28,29]. These antigenic loops have also been identified in the hemagglutinin of H5N1 viruses by structural determination [30]. Comparison of the amino acid sequences of numerous H5N1 hemagglutinins shows genetic drift in these regions, and the introduction of a potential glycosylation site in the 150s loop of virus isolated in Vietnam during 2004. Micro-neutralization assays using A/Vietnam/1203/04 and A/Hong Kong/213/03, designed to select for antibodies not dependant on the 150s loop for binding, identified two mAb (VN04-2 and VN04-3) which showed similar levels of neutralization against both H5N1 viruses.

Since the ultimate goal is to identify antibodies which can be used as passive antibody prophylaxis against human infection with H5N1 virus, we humanized the antibody prior to efficacy studies by replacing the constant regions of the mouse mAb with those of human IgG1 heavy chain and kappa light chain. The chimeric antibodies retained the specificity of the parent mouse mAbs. Humanization is a complex issue (excellently reviewed [31]), designed to overcome the problems associated with use of mouse mAbs in humans, such as human anti-mouse-antibody (HAMA) responses that limits their repeated use: the extent of humanization versus the reduction of HAMA response is still an open question. Some human anti-chimeric antibody responses have been observed, however some human antibodies against more completely humanized mouse mAb have also been observed. Nevertheless, antibodies humanized by both methods used therapeutically and repetitively in the same individual are evident and the question of which is the best method still remains open. Perhaps the ultimate test will be in phase 1 clinical trials where the development of human antibodies against the introduced humanized antibody can be experimentally determined.

As a prelude to the possibility of testing the clinical application of these antibodies in human trials, their efficacy in an animal model needs to be determined. To this end, protection against lethal virus infection in a C57BL/6 mouse model both in a prophylactic and therapeutic context was performed. VN04-2-huG1 performed better *in vivo *than VN04-3-huG1 as complete protection against virus challenge with very little sign of disease with the lowest tested antibody concentration of 1 mg/kg was observed. This level of VN04-2-huG1 was not as effective therapeutically when used one or three days after infection, as protection was reduced to 80% and significant signs of disease were evident. However increasing the dosage of the antibody did restore complete protection and limit illness. As expected, a correlation of antibody dosage required for effective treatment versus the time of treatment after infection was evident, as more antibody was required to achieve similar therapeutic efficacy when the antibodies were introduced three days after infection compared to one day post infection. This result supports the recent study showing therapeutic efficacy of equine hyperimmune globulin F(ab')2 against H5N1 virus one day post infection [20], and may allow for extension of their therapeutic potential to three days post infection. The efficacy of VN04-2-huG1 both as prophylaxis and therapy suggests that this antibody should be considered for further evaluation as a passive antibody prophylaxis against H5N1 virus infection for use in humans.

A potential drawback to the use of passive antibodies is the current high cost of large scale antibody production. This raises the costs of treatments utilizing antibodies, such as for RSV infection and autoimmune disease, to several thousands of dollars per treatment. It is also worthy of note that these antibodies are among the first commercially available antibodies for clinical use, a factor which contributes to the high cost and also that the amount of antibody administered is very high. Should an influenza pandemic arise, the increased burden on infrastructure as well as the likely effect on tourism and international trade would have a large impact on the economies of many countries. Therefore, the widespread use of protective antibodies to mitigate virus spread would become a governmental decision, as faster economic recovery through decisive and rapid containment of a pandemic would make the cost of the antibodies negligible when compared to the cost of a prolonged pandemic. However, antibody dosage for every member of the population may not be necessary. Recent studies examining containment strategies for pandemic influenza using a combination of geographically targeted prophylaxis with antiviral drugs and social distancing have suggested that a stockpile of 3 million doses of antiviral may be sufficient to contain an emerging pandemic at the source [32,33]. Given the efficacy of VN04-2-huG1, it would not be unwarranted to suggest that passive antibody therapy with these antibodies could be used in a similar manner to antiviral drugs in this model, drastically reducing the amount of antibody needed for stockpiling to an achievable level.

While the antibodies described here are specific for the hemagglutinin of H5N1 viruses of the Z-genotype circulating in 2003/2004, the recent emergence of multiple sublineages of H5N1 virus in Asia, some of which are antigenically distinct to those used here [26], raises the possibility that a future pandemic influenza may escape the protective effect of these antibodies. Indeed, determination of the epitope of VN04-2-huG1 using the HI data presented here and that detailed in the abovementioned study identified the 140s antigenic loop as responsible for antibody binding and suggests a requirement for lysine at position 140. However it should be noted that all of the HI assay negative strains contain a mutation at residue 94. While this residue faces inside the hemagglutinin trimer and would not be exposed for antibody binding, it may still have an effect on the performance of the HI assays, as was the case with mutation of a serine at position 223 to asparagine [22], raising the possibility that the negative result for the HI assays may be more a limitation of the assay rather then the ability of the antibody to bind to the virus. In either case, mutations within the antigenic loops of hemagglutinin have been shown to effect antibody binding and neutralization [34,35], and more recent isolates of H5N1 virus have shown mutation from those circulating in 2004 within these antigenic regions.

The effect of the mutations on the protective efficacy of VN04-2-huG1 for influenza A H5N1 virus currently circulating would have to be determined experimentally. If indeed the 140s antigen loop is a major determinant of the epitope for VN04-2-huG1, then a future pandemic strain may escape the protection afforded by this antibody. To combat such an outcome, a panel of proven protective antibodies could be established against multiple antigenic loop variants. Although, the question of mutation within the antigenic regions of a future pandemic strain allowing escape would still be an important issue. Ideally, a neutralizing antibody whose epitope determinants are not within the antigenic loops and less prone to mutation may somewhat overcome this issue, but the ability to identify such an antibody may not be possible. Nevertheless, we have shown here the 'proof of principle' that passive antibody therapy can be an effective tool for both prophylaxis against- and treatment of- highly pathogenic H5N1 influenza virus, providing the immediate immunity needed which combined with social distancing could limit the transmission of H5N1 virus to others and contain a future influenza pandemic.

## Competing interests

The author(s) declare that they have no competing interests.

## Authors' contributions

BJH participated in the design of the study, carried out the epitope mapping and drafted the manuscript. ACMB performed the animal experimentation. APCL humanized and produced the antibodies and performed the immunoassays. AW synthesized the cDNA and performed the virus neutralization and HI assays. EEO and RJW conceived the study. All authors read and approved the final manuscript.
